# Test-retest reliability and construct validity of trunk extensor muscle force modulation accuracy

**DOI:** 10.1371/journal.pone.0289531

**Published:** 2023-08-17

**Authors:** John R. Gilliam, Ahyoung Song, Pradeep K. Sahu, Sheri P. Silfies

**Affiliations:** 1 Department of Exercise Science, University of South Carolina, Columbia, South Carolina, United States of America; 2 Physical Therapy Program, University of South Carolina, Columbia, South Carolina, United States of America; Adnan Menderes Universitesi, TURKEY

## Abstract

Low back pain is associated with changes in trunk muscle structure and function and motor control impairments. Voluntary force modulation (FM) of trunk muscles is a unique and under-investigated motor control characteristic. One of the reasons for this paucity of evidence is the lack of exploration and publication on the reliability and validity of trunk FM protocols. The purpose of this study was to determine the within- and between-day test-retest reliability and construct validity for trunk extensor muscle FM. Twenty-nine healthy participants were tested under three FM conditions with different modulation rates. Testing was performed on a custom-built apparatus designed for trunk isometric force testing. FM accuracy relative to a fluctuating target force (20–50%MVF) was quantified using the root mean square error of the participant’s generated force relative to the target force. Reliability and precision of measurement were assessed using the Intraclass Correlation Coefficient (ICC), standard error of measurement (SEM), minimal detectable difference (MDD_95_), and Bland-Altman plots. In a subset of participants, we collected surface electromyography of trunk and hip muscles. We used non-negative matrix factorization (NNMF) to identify the underlying motor control strategies. Within- and between-day test-retest reliability was excellent for FM accuracy across the three conditions (ICC range: 0.865 to 0.979). SEM values ranged 0.9–1.8 Newtons(N) and MDD_95_ ranged from 2.4–4.9N. Conditions with faster rates of FM had higher ICCs. NNMF analysis revealed two muscle synergies that were consistent across participants and conditions. These synergies demonstrate that the muscles primarily involved in this FM task were indeed the trunk extensor muscles. This protocol can consistently measure FM accuracy within and between testing sessions. Trunk extensor FM, as measured by this protocol, is not specific to any trunk muscle group but is the result of modulation by all the trunk extensor muscles.

## Introduction

Trunk extensor muscles including the superficial erector spinae (longissimus thoracis and iliocostalis lumborum) and deep and superficial multifidi are contributors to the stability of the spine and to the coordination of trunk and limb movements [1]. Changes in trunk extensor muscle structure including muscle atrophy, fiber-type transitioning, fibrosis, and fatty infiltrate [2–7] and impairments in muscle function encompassing strength, rate of force production, and endurance are well documented in persons with low back pain (LBP) [8–13]. Literature suggests impairments in neuromuscular control are common in LBP and may persist even after the resolution of symptoms [14]. Motor control is the process by which the nervous system controls posture and task-associated movement by integrating sensory, motor, and cognitive systems [15]. Motor control deficits such as delayed trunk muscle reflex response, impaired motor planning, and altered activation patterns are associated with LBP and can adversely impact movement patterns [2, 15–20]. While many variables representing motor control function have been researched, force modulation (FM) is a unique motor control characteristic with limited investigation as an underlying mechanism of movement impairment in individuals with LBP.

Muscle FM requires the dynamic interplay between sensory inputs and motor outputs occurring at multiple levels of the nervous system and is defined as the capacity of the neuromuscular system to generate coordinated, accurate, and smooth force output [21, 22]. Voluntary muscle contraction occurs as a result of alpha and gamma motor neuron co-activation and is associated with increased muscle spindle and Golgi tendon organ discharge. In some afferent nerve endings, discharge grows linearly with the force of contraction [23–28]. The additional information provided by these structures is proposed to provide more flexibility within the neuromuscular system to grade force production [25]. Trunk muscle FM is critical for human movement and function. Walking, carrying, pushing, pulling, and many other fundamental patterns of movement require fluctuating, submaximal FM of an array of trunk muscles working in concert to achieve the desired movement outcome [29–31]. Experimentally, FM accuracy can be quantified by measuring the difference between submaximal force output generated by a participant and a static or dynamic target force, with a large difference suggesting less accuracy [22, 26, 32, 33]. In the lower extremity, diminished FM accuracy has been associated with key functional outcomes such as history of falling [34], walking endurance [35], stair climbing [36], and performance on a hop test following anterior cruciate ligament reconstruction [26, 32, 33]. Few studies have investigated trunk FM accuracy in individuals with LBP, but each demonstrates that persons with LBP exhibit impaired FM accuracy compared with back-healthy subjects.[22, 37, 38] Further, diminished FM accuracy was associated with LBP-related disability [22].

One reason trunk muscle FM has been understudied is a lack of valid and reliable protocols to assess this construct. Only two trunk muscle FM reliability studies were discovered [39, 40]. Hadizadeh et al. [39] evaluated the reliability of FM error in nine healthy participants, and reported good to excellent intraclass correlation (ICC) values. Similarly, Reeves et al. [40] demonstrated excellent between-day ICC values and small standard error of the measurement (SEM) values in a small study (n = 10) of healthy participants. Other literature to date has reported only reliability from non-peer reviewed/unpublished studies [22, 41] or failed to address the validity and reliability of their FM protocols altogether [37, 42]. Little is known about how different factors such as FM intensity range, modulation form, and modulation rate influence the reliability of FM protocols. Further, we do not know which trunk and hip motor control strategies contribute to the forces generated in these protocols. To the authors’ knowledge, no studies have published data on surface electromyography (sEMG) of the trunk and hip muscles during a trunk extensor FM task. While experimental protocols and testing equipment are designed to bias force generation to the lumbar extensor muscles, the assumption that the lumbar extensor muscles are the primary muscles responsible for FM in these tasks remains untested. Given that the neuromuscular system of the trunk is complex, redundant, and with muscles crossing multiple segments, many possible combinations of muscle activity result in a movement outcome. It is important to understand the contributions of known groups of trunk and hip muscles to establish the construct validity of “trunk extensor” FM protocols. Only if we determine which muscles are involved and to what extent they contribute to FM, can we draw accurate conclusions about the findings of FM protocols and potential impairments in individuals with LBP.

The purpose of this study was: First, to determine the within- and between-day test-retest reliability of trunk extensor FM accuracy using a protocol with three conditions utilizing different rates of FM in healthy individuals. And second, to evaluate the construct validity of “trunk extensor FM” by using sEMG and non-negative matrix factorization (NNMF) to describe the muscle activation patterns underlying FM and the effect of the modulation rate on those neuromuscular strategies. We hypothesized that our FM protocol would demonstrate good to excellent within- and between-day test-retest reliability and that our sEMG and NNMF results would support the construct of “trunk extensor” FM.

## Materials and methods

### Participants

A convenience sample of 29 healthy participants was recruited from the University of South Carolina between August 2020 and April 2021. English-speaking adults ages 18–35 years old were included; this range was selected to ensure skeletal maturity with a reasonably low prevalence of osteoarthritis or osteopenia. Participants were excluded if they had a history of back pain (defined as missing three days of work or recreational activity or resulted in seeking attention from a healthcare professional), a history of concussion within the last six months, a diagnosis of fibromyalgia, rheumatoid arthritis, or chronic fatigue syndrome, previous spine or hip surgery, or any current injury to the lower extremities. Seven additional participants completed the protocol with simultaneous recording of trunk sEMG data to assess muscle activation patterns during the task. Written informed consent was obtained from each participant prior to testing. The study protocol received approval from the University of South Carolina internal review board (ID: Pro00102363).

### Testing apparatus

**Fig 1:**
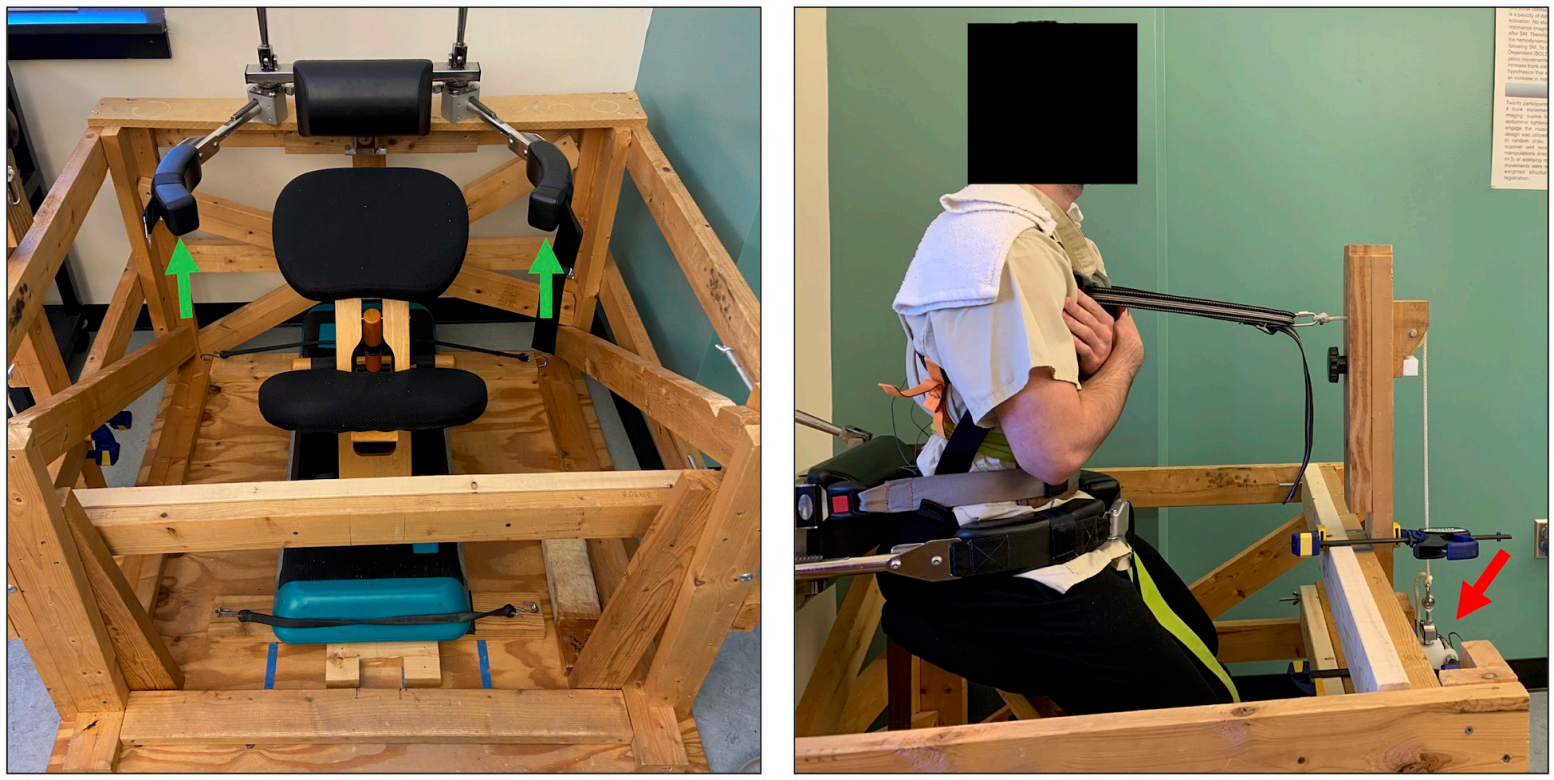
Testing apparatus and experimental setup. Left: Balans chair inside custom wood-built testing apparatus. In this setup, the pelvis is stabilized by securing padded arms (green arrows) around the anterolateral aspect of the pelvis and locking the pads into place. Right: Sagittal view of participant set up for testing with the pelvis locked into place. The tension load cell (red arrow) measuring extensor force generated by the participant is secured to a chest harness at the height of the xiphoid process.

A custom-built wood cage with a Balans chair in the middle was constructed for trunk muscle force testing (Fig 1, left). To minimize hip extension movement, padded stabilization arms were used to secure the position of the pelvis on the chair. For additional pelvic stabilization, a padded mobilization belt was wrapped around the anterior pelvis and seat of the chair. A trunk climbing harness is attached to the participant by first tightening anterior straps followed by posterior straps that wrap around the mid trunk. A separate belt is secured from the anterior harness to a 500 lb. tension load cell and amplifier (MPL- 500, Transducer Techniques, Temecula, CA) attached to the wood frame of the testing apparatus. The load cell is attached via a single pulley system aligned with the height of the participant’s xiphoid process (Fig 1, right). Once the participant was seated and appropriately adjusted within the testing apparatus, trunk flexion angle was measured using an inclinometer placed over the T3 spinous processes and hip flexion angle was measured using a goniometer; these angles were replicated for consistency in repositioning during the retest protocol. A large force target is presented on a 32-inch monitor at eye level to permit visualization of the target force and the feedback of the participant-generated force.

### Data collection

**Fig 2:**
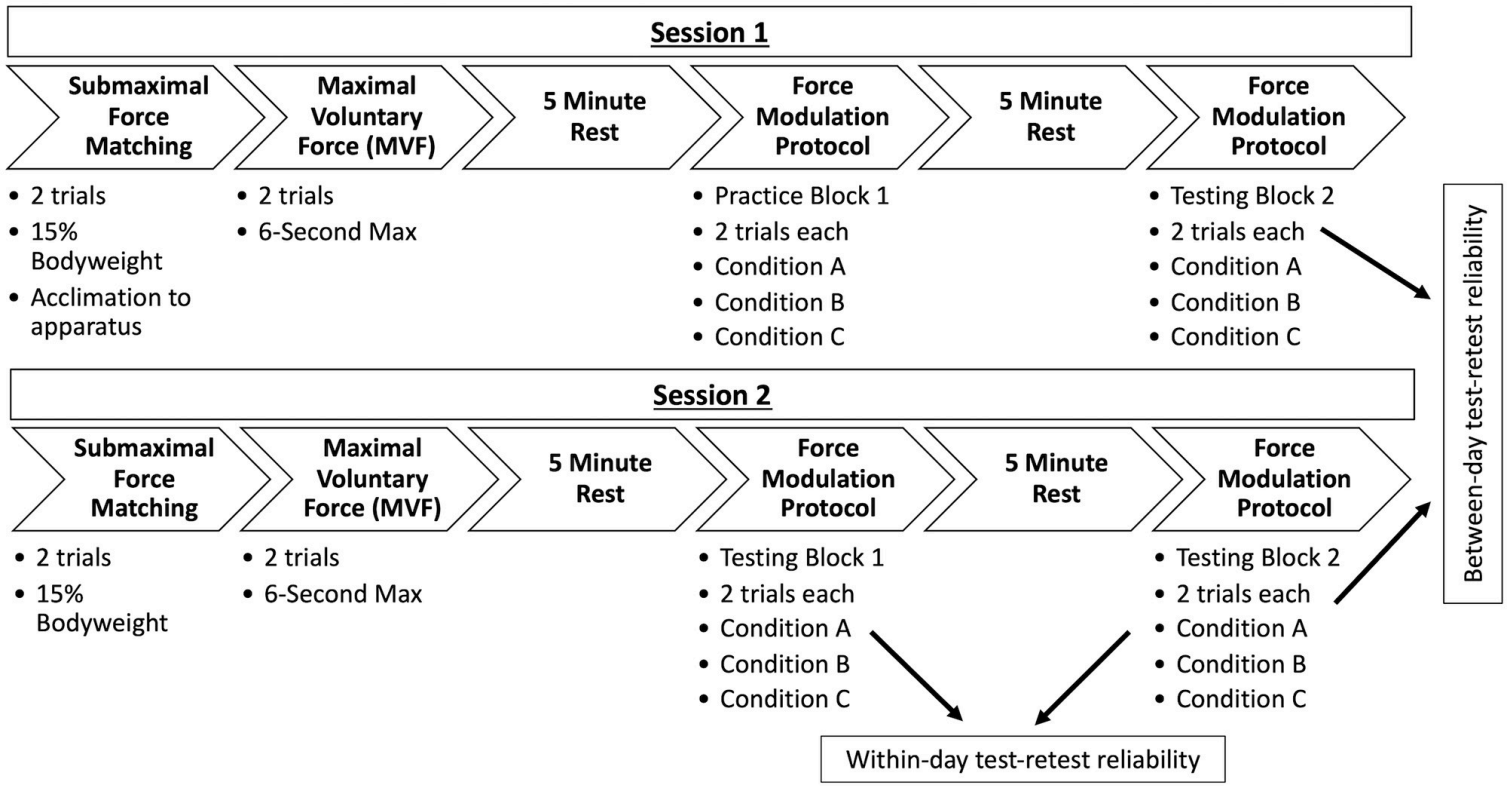
Schematic of the study protocol and testing timeline. Participants completed two testing sessions separated by 2–7 days. Within-day reliability data was calculated using the testing blocks in session 2. Between-day reliability as calculated from testing block 2 of each session.

Prior to testing, participants filled out the Minnesota Leisure Time Physical Activity Questionnaire (MLTPAQ) to evaluate any impact of physical activity level on FM accuracy. To assess the contribution of known muscle groups to FM, wireless sEMG data were collected on seven participants using active, bipolar, differential electrodes (Delsys, Trigno, Natick, MA). Sensors were applied over five trunk and hip muscles bilaterally: thoracic erector spinae (TES), lumbar erector spinae (LES), lumbar multifidus (LM), gluteus maximus (GMX) and external oblique (EO). Electrode placement was standardized and consistent with prior literature [43–45]. To normalize sEMG values we collected individual muscle maximal voluntary isometric contractions (MVICs). Details regarding sEMG sensors, sensor placement, and MVIC testing positions can be found in S1 Appendix. Force data from the tension load cell and digital to analog converted sEMG data were simultaneously collected at 1600 Hz using a custom LabVIEW program (version 8.6, National Instruments, Austin, TX). Participants repeated two identical testing sessions, 2–7 days apart. Each testing session included two submaximal force matching trials, two maximal voluntary force (MVF) trials, and two testing blocks with four total trials for each of FM conditions (Fig 2).

### Maximum voluntary force protocol

**Fig 3:**
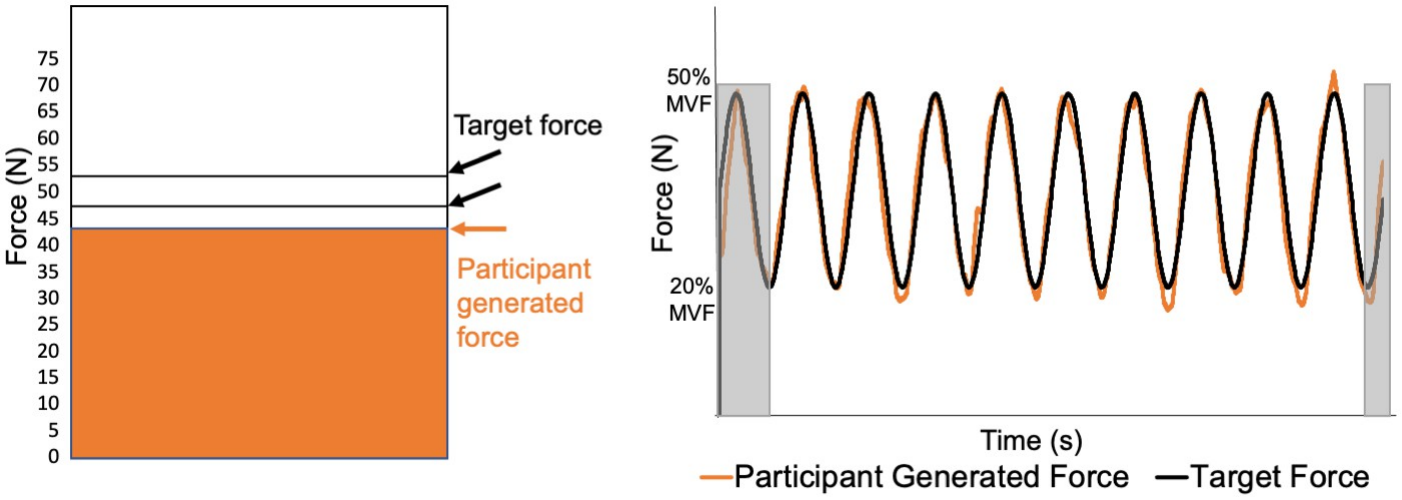
Force modulation feedback and time series. Left: A recreation of visual feedback located on monitor 1m in front of the participant. The two horizontal lines representing the target force fluctuate up and down while the participant tries to keep their generated force (orange) between these two lines. Right: Time series plot of target force (black line) fluctuating between 20–50% maximum voluntary force and the participant-generated force (orange line). The first repetition (sine wave) and the ascending portion of the last repetition in each trial were eliminated to allow the required time to develop or drop below the lower force boundary (gray boxes).

All participants trunk extensor isometric muscle force production was determined using MVF trials. Prior to max trials, two 6-second submaximal isometric force matching were completed as a warmup to familiarize individuals with the apparatus. For submaximal trials, participants received visual feedback in real-time of the force they generated and were instructed to match a target force (15% body weight) by keeping their force production between two horizontal lines (force target ± 5 Newtons (N)) presented on the monitor (Fig 3, left). Participants then performed two 6-second MVF trials with a 1-minute rest break between trials. The maximum force (N) achieved within each MVF trial was recorded, and the average considered the trunk extensor MVF. Average MVF was used to determine the individual range (%MVF) of modulation during the FM conditions. No visual feedback or verbal encouragement was provided for MVF trials. Ratings of perceived exertion (RPE, 6–20 scale) [46, 47] were collected following each MVF trial. Submaximal force matching and MVF testing were completed during both testing sessions.

### Force modulation protocol and conditions

The FM intensity range was 20%-50% MVF. This target range was selected based on trunk extensor muscle contraction intensities utilized during activities of daily living [29, 30]. The target force range fluctuated, increasing and decreasing, according to a sine wave function (Fig 3, right). Three FM conditions (conditions A, B, and C) were performed in order of increasing modulation frequency. Condition A consisted of five full repetitions (increasing and decreasing) over 60 seconds (.083 Hz) [22]. Condition B included ten repetitions over 60 s (.167 Hz); and condition C, ten repetitions over 45 s (.222 Hz). In each testing block, each condition was performed twice with a 60 s break between trials and conditions. The participants were provided a visual target using a vertical box with two horizontal lines moving vertically inside the box to indicate the target force (Fig 3, left). Following the performance of each condition two times, a longer rest break of 5 minutes was provided. Ratings of perceived exertion (RPE, 6–20 scale) were collected following each trunk extensor FM condition [47]. Previous unpublished work in our lab indicated that a single initial testing block was necessary to familiarize the participant with the task and stabilize FM accuracy values; therefore, data from testing block 1 in session 1 was considered a practice block and was not used to calculate reliability statistics or for muscle synergy analysis.

### Data processing

FM accuracy relative to the target force was quantified using the root mean square error (RMSE) of the participant’s generated force relative to the target force, with higher scores indicating poorer performance. Mean FM accuracy was calculated as the average RMSE value of the two trials in each testing block for each condition.

sEMG data from a single testing session on 7 participants underwent heart rate stripping using a fast independent component analysis [48, 49] within a custom LabVIEW program (v8.6; National Instruments, Austin, TX). Raw sEMG signals were demeaned, filtered [root mean square (RMS), Bartlett, time constant (Tc) = 250 ms], amplitude normalized to individual muscle MVICs [mean amplitude of middle 3–5 seconds of MVIC trial data], and time normalized by interpolation to 500-data points representing a single, full repetition (ascending and descending sine wave). sEMG signals used for normalization were visually inspected to confirm no abrupt changes or spikes in amplitude. The mean amplitude of muscle activations at minimum (20% of MVF) and maximum target forces (50% of MVF) from time- and amplitude-normalized sEMG were calculated for each condition, and then group-averaged amplitude values were computed.

### Muscle synergy analysis

**Fig 4:**
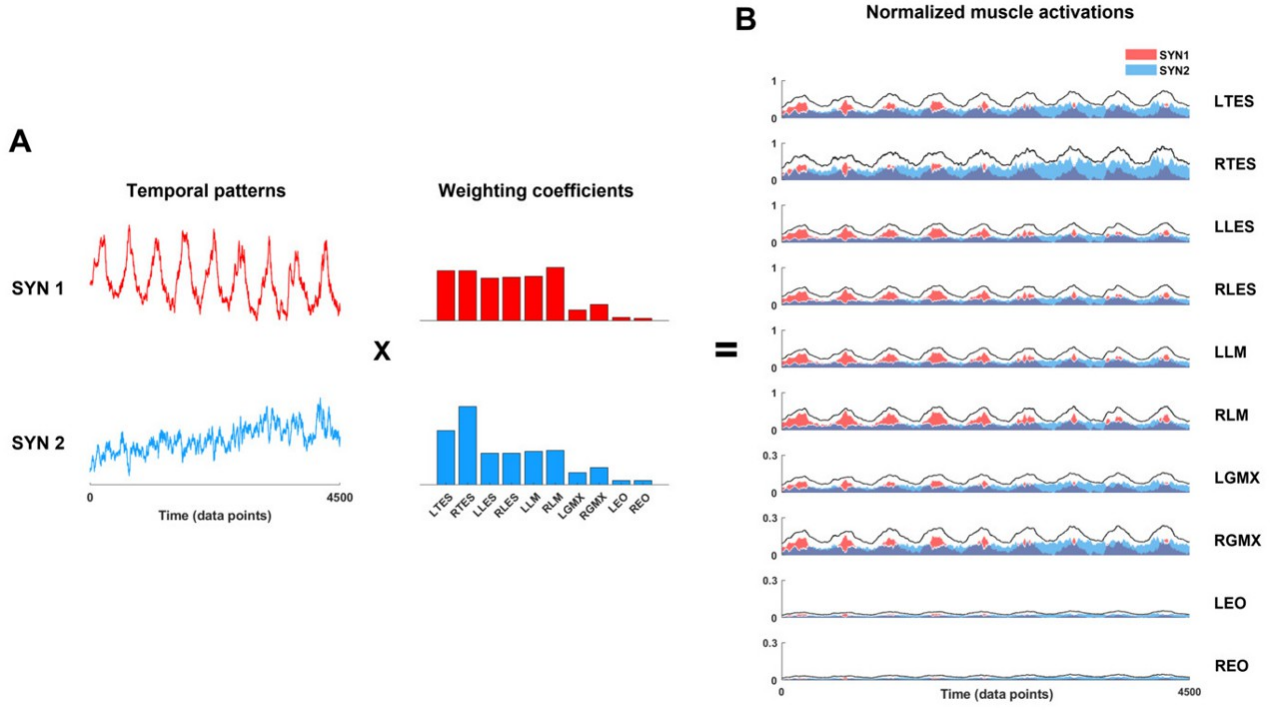
**A**. Example of muscle synergies that were extracted from a single subject’s time- and amplitude-normalized EMG during condition B (nine repetitions of task). Temporal patterns multiplied by weighting coefficients from the extracted synergies recreate the normalized muscle activations. **B**. Individual muscle activation is the sum of the contribution of each synergy. Y-scale of normalized EMG where 1 represents 100% MVIC. SYN, synergy; LTES, left thoracic erector spinae; RTES, right thoracic erector spinae; LLES, left lumbar erector spinae; RLES, right lumbar erector spinae; LLM, left lumbar multifidus; RLM, right lumbar multifidus; LGMX, left gluteus maximus; RGMX, right gluteus maximus; LEO, left external oblique; REO, right external oblique.

Non-negative matrix factorization was applied to identify the underlying muscle synergies and to characterize the interaction ofmuscle amplitude and timing used to modulate the force. For each participant and FM condition (A, B & C), pre-processed (time- and amplitude normalized) sEMG (*E*) was decomposed into muscle synergies using the low-rank approximation (*Ê*):

Ê=W×H+e, (1)

where *Ê* (*m* × *t* matrix, where *m* is the number of muscles and *t* is the number of data points) is an approximation of muscle activity (*E*), based on the linear combination of weighting coefficients *W* (*m* × *n* matrix, where *n* is the number of synergies extracted) and activation patterns *H* (*n* × *t* matrix) (Fig 4). Weighting coefficients and activation patterns were estimated by minimizing the root mean square residual error between *E* and *Ê*. The variance of the muscle activity accounted for (VAF, *r*^*2*^) by extracted synergies was calculated using the formula:

$$r^{2}=1-\;\text{sum}\;\text{of}\;\text{squared}\;\text{errors}/\text{total}\;\text{sum}\;\text{of}\;\text{squares},$$ (2)

The best linear fit method [50] was used to determine the number of synergies extracted with the residual mean square error (RMSE) threshold of 0.0001. A factor clustering technique was utilized to compare the similarity of muscle synergies across participants. The best matching scalar product of normalized weighting coefficients was used to calculate the similarity between synergies [51]. Group averaged weighting coefficients served as the initial set to compare vectors in each individual and for each condition using the best matching scalar product. Each possible combination was compared between each participant’s and the group-averaged synergy set. If two synergies from an individual matched with one synergy in the group-averaged template with the scalar products >0.6, those two synergies are considered as the same synergy patterns. Group averaged weighting coefficients and temporal activation patterns were then computed by averaging the synergy with the highest scalar product from individuals. All sEMG analyses were conducted in MATLAB 2022b (Mathworks, Natick, MA, USA).

### Statistical analysis

Statistical analyses were conducted using Microsoft Excel (Microsoft Corporation, Redmond, WA.), MATLAB 2022b (Mathworks, Natick, MA, USA), and SPSS (IBM SPSS version 24, Armonk, NY). Demographic data were checked for normality and demographic differences between subjects used in reliability and validity analyses were assessed using t- and chi-square tests. Within- and between-day test-retest reliability of trunk extensor FM accuracy across three conditions were investigated by calculating intraclass correlation coefficients (ICC_2,k_) (two-way mixed effects, absolute agreement, multiple measurements) [52]. A single participant had missing RMSE data for retest values for conditions A and C. Based on previous literature, we categorized ICCs < 0.40 as poor reliability, 0.40 ≤ ICC < 0.75 as good reliability, and ICC ≥ 0.75 as excellent reliability [53]. SEM and minimal detectable difference (MDD_95_) were also calculated for within-and between-day comparisons for each condition using the following formulas:

$$\text{SEM}={\text{SD}}_{\text{pooled}}\sqrt{(1}-ICC)$$ (3)

$${\text{MDD}}_{95}=1.96\left(SEM\right)\text{X}\sqrt{\left(2\right)}$$ (4)

Bland-Altman Plots were constructed for visual inspection to assess for systematic bias and evaluate the limits of agreement between test and retest [54]. Self-report physical activity level from the MLTPAQ was assessed for normality. To determine if fitness level influenced the protocol, we assessed the correlation between MLTPAQ score and the mean FM error across the three testing blocks (excluding the practice block) using a Spearman’s rank correlation coefficient. To determine if fatigue influenced the testing protocol, we assessed RPE data. RPE data were visually checked for normality using histograms. Differences in average RPE over the four testing blocks were assessed using a repeated measures ANOVA for each condition. If the overall F test with Greenhouse-Geisser adjustment was significant, pairwise comparisons were conducted with Bonferroni correction. Differences in average RPE scores across the three FM conditions were assessed using a one-way ANOVA. Normalized muscle activations were combined across participants and reported as the mean and standard deviation for the three conditions. Once muscle synergy patterns were identified for each experimental condition, synergies were compared across conditions to determine if they represented the same synergy using the scalar product, with >0.9 value indicating high similarity between synergies.

## Results

### Participant demographics

Participants were relatively young and active with an average BMI in the healthy range. No differences were observed between groups for age, BMI, or physical activity level (MLTPAQ). Female participants made up a little more than half the sample, and the frequency of female participants was not different between groups (Table 1).

**Table 1:** Characteristics of study participants.

Participant characteristics	Reliability (n = 29)	Validity (n = 7)	Test for Group Diff.
Age (years)	24.9 (4.0)	24.3 (1.0)	p = 0.83
Gender*	15F (52%)	4F (57%)	p = 0.73
Body mass index (BMI)	24.9 (4.0)	24.1 (3.9)	p = 0.64
MLTPAQ	12105 (12196)	11606 (5772)	p = 0.97

Data presented as mean (standard deviation). MLTPAQ: Minnesota Leisure Time Physical Activity Questionnaire

### Test-retest reliability

**Fig 5:**
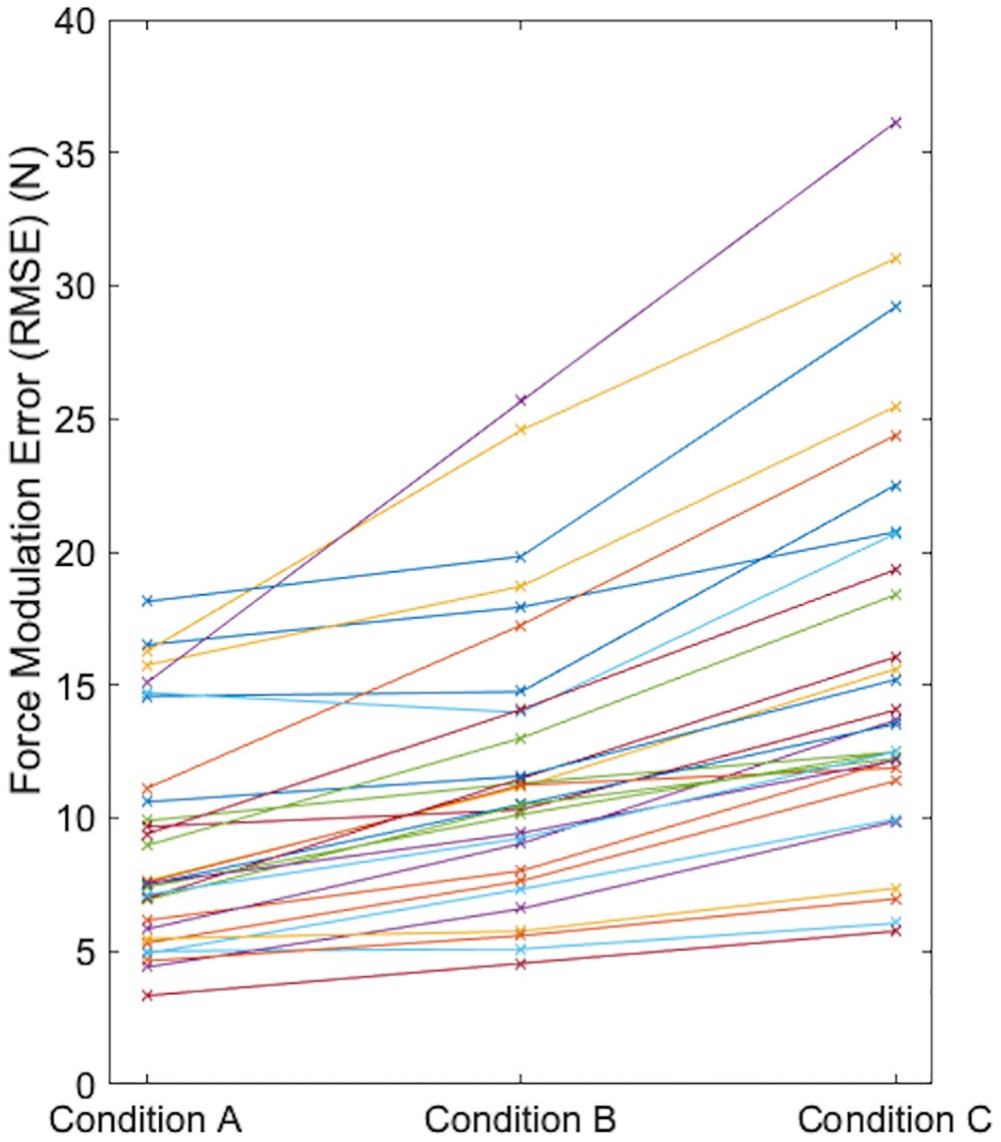
Force modulation accuracy of the individual subjects across conditions. Each colored line represents an individual subject’s mean force modulation error (RMSE) from the testing blocks across the three conditions. Generally, error increased from protocol A to B and again from B to C. Faster rates of force modulation also resulted in greater between-subject variability. RMSE, root mean square error.

Conditions with faster rates of FM revealed higher FM error and greater between-subject variability in task performance (Fig 5). Within-day test-retest reliability for measuring FM accuracy was excellent across the three conditions and ranged from 0.923 to 0.979 ICC_(2,2)_ with MDD_95_ ranging from 2.4 to 3.2 N with the smallest MDD_95_ values associated with condition B (Table 2). Between-day test-retest reliability was also excellent with a slightly larger ICC_(2,2)_ range of 0.865 to 0.976 with MDD_95_ ranging from 3.4 to 4.9 N. For between-day measurements, participants demonstrated the smallest MDD_95_ during condition C.

**Table 2:** ICC, SEM, and MDD values for test-retest reliability of trunk extensor force modulation protocol.

Within-day test-retest reliability*					
	Force Modulation Accuracy (RMSE)		Reliability		
Condition	Mean (SD) Block 1	Mean (SD) Block 2	ICC_(2,2)_ (95% CI)	SEM	MDD_95_
A	9.41 (4.19)	8.79 (4.16)	0.923 (0.834–0.964)	1.2	3.2
B	12.10 (5.49)	11.62 (5.53)	0.975 (0.947–0.988)	0.9	2.4
C	16.27 (7.78)	15.95 (7.94)	0.979 (0.955–0.990)	1.4	3.2
Between-day test-retest reliability**					
	Force Modulation Accuracy (RMSE)		Reliability		
Condition	Mean (SD) Session 1	Mean (SD) Session 2	ICC_(2,2)_ (95% CI)	SEM	MDD_95_
A	9.34 (5.57)	8.70 (4.12)	0.865 (0.716–0.937)	1.8	4.9
B	11.96 (5.80)	11.62 (5.53)	0.928 (0.846–0.966)	1.5	4.2
C	16.19 (7.58)	16.06 (8.06)	0.976 (0.947–0.989)	1.2	3.4

*Within-day test-retest reliability: comparing average force modulation accuracy of 2 trials from session 2 block 1 and 2 trials from session 2 block 2.

**Between-day test-retest reliability: comparing average force modulation accuracy of 2 trials from session 1 block 2 and average of 2 trials from session 2 block 2.

Mean, SD, SEM and MDD values are presented in root mean square error (RMSE) values.

SD: Standard deviation, ICC: intraclass correlation coefficient, 95% CI: 95% confidence interval, SEM: Standard error of measure, MDD: minimal detectable difference

**Fig 6:**
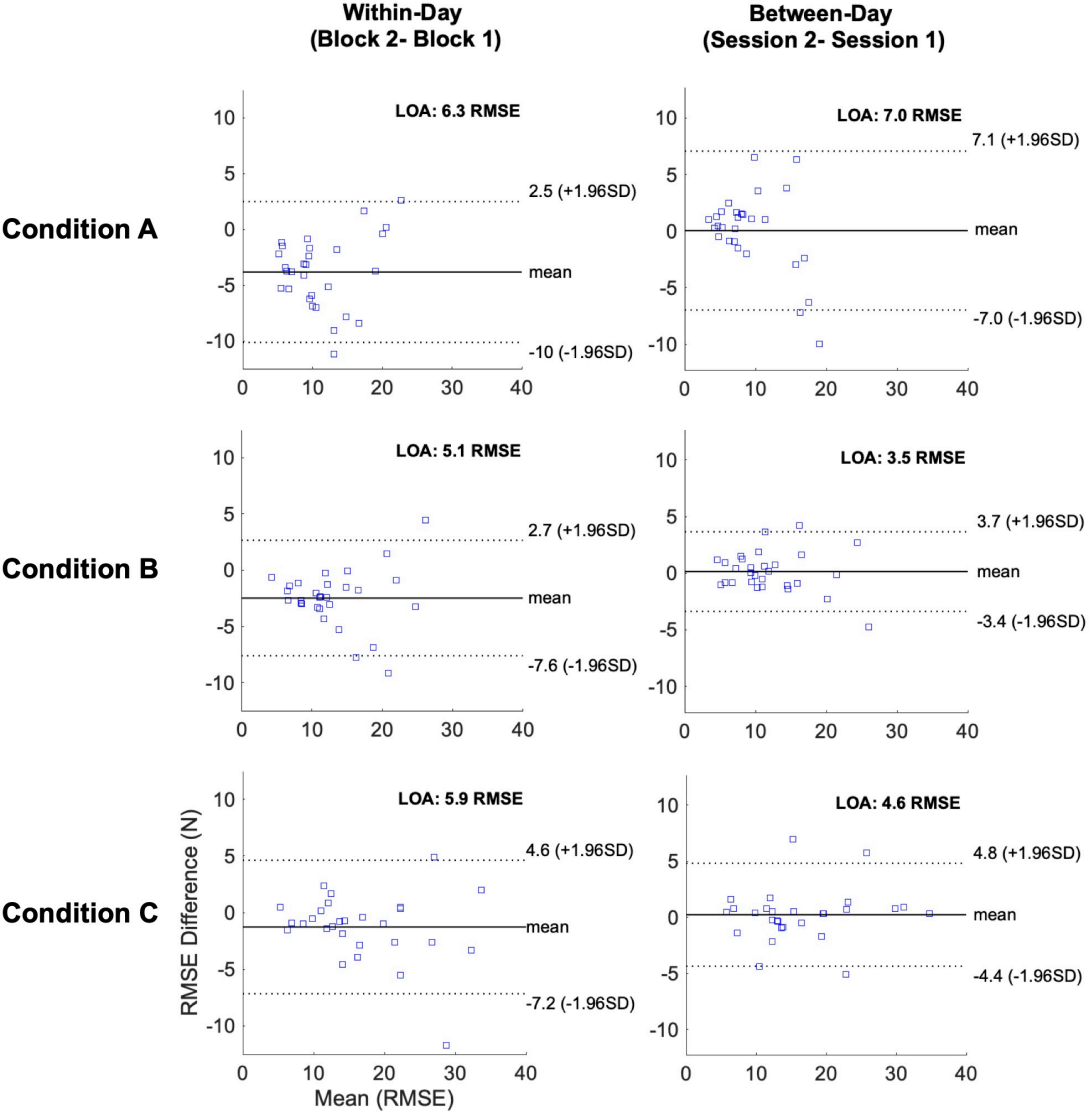
Bland Altman plots for within- and between-day comparisons of RMSE. Limits of agreement (LOA) include 95% of differences between the two measurements. Systematic bias is observed where individuals who demonstrated higher mean RMSE values tended to have larger differences between measurements. This is most evident in condition A. RMSE: root mean square error, SD: standard deviation.

Bland-Altman plots demonstrate good within- and between-day agreement across all three conditions. Bland-Altman plots also reveal heteroscedasticity where individuals with higher RMSE values (less accuracy) tended to have less consistent performance. Bias is observed as a greater between-subject variance in difference score (further from the average mean difference) as you move further to the right along the x-axis (Fig 6).

### Physical activity level & rating of perceived exertion

Spearman’s rank correlation coefficients revealed that physical activity level measured by MLTPAQ score was correlated with mean FM error (Spearman’s rho = -.287 to -.367; *p* = .027-.069) where higher levels of physical activity were associated with lower error scores or better FM accuracy. Depending on the condition, physical activity level accounted for 8–13% of the variance in FM error. Differences in RPE were found for conditions A and B over the four testing blocks (Table 3). Pairwise comparisons revealed that for condition A: session 2 block 1 was significantly different from the two testing blocks on session 1, but not different than block 2 on the same day. For condition B: session 2 block 1 was different from session 1 block 2, but not the other testing blocks. RPEs in condition C over the testing blocks were not different, but, like conditions A and B, condition C demonstrated a slight decrease in RPE on session 2 block 1. There were no differences in average RPE across the three conditions (F = .281, *p* = .756).

**Table 3:** Mean rate of perceived exertion (RPE 6–20) for conditions A, B, and C across four testing blocks.

Condition	Session 1 Block 1	Session 1 Block 2	Session 2 Block 1	Session 2 Block 2	Mean (4 Blocks)
A	12.7 (2.6)	12.4 (1.7)	11.1 (2.2)*	11.6 (2.6)	12.0 (1.8)
B	12.6 (2.0)	12.8 (1.9)	11.8 (2.3)^†^	12.0 (2.4)	12.3 (1.9)
C	12.5 (1.7)	12.6 (1.8)	11.8 (2.4)	11.9 (2.4)	12.2 (1.8)

Data reported as mean (standard deviation)

*For condition A: Day 2 Block 1 was significantly lower RPE scores (p < .05) than Day 1 Block 1 and Day 1 Block 2, but not different from Day 2 Block 2.

^†^For condition B: Day 2 Block 1 was significantly lower RPE scores (p < .05) than Day 1 Block 2, but not different from Day 1 Block 1 and Day 2 Block 2.

### Mean sEMG amplitude at minimum and maximum target forces

**Fig 7:**
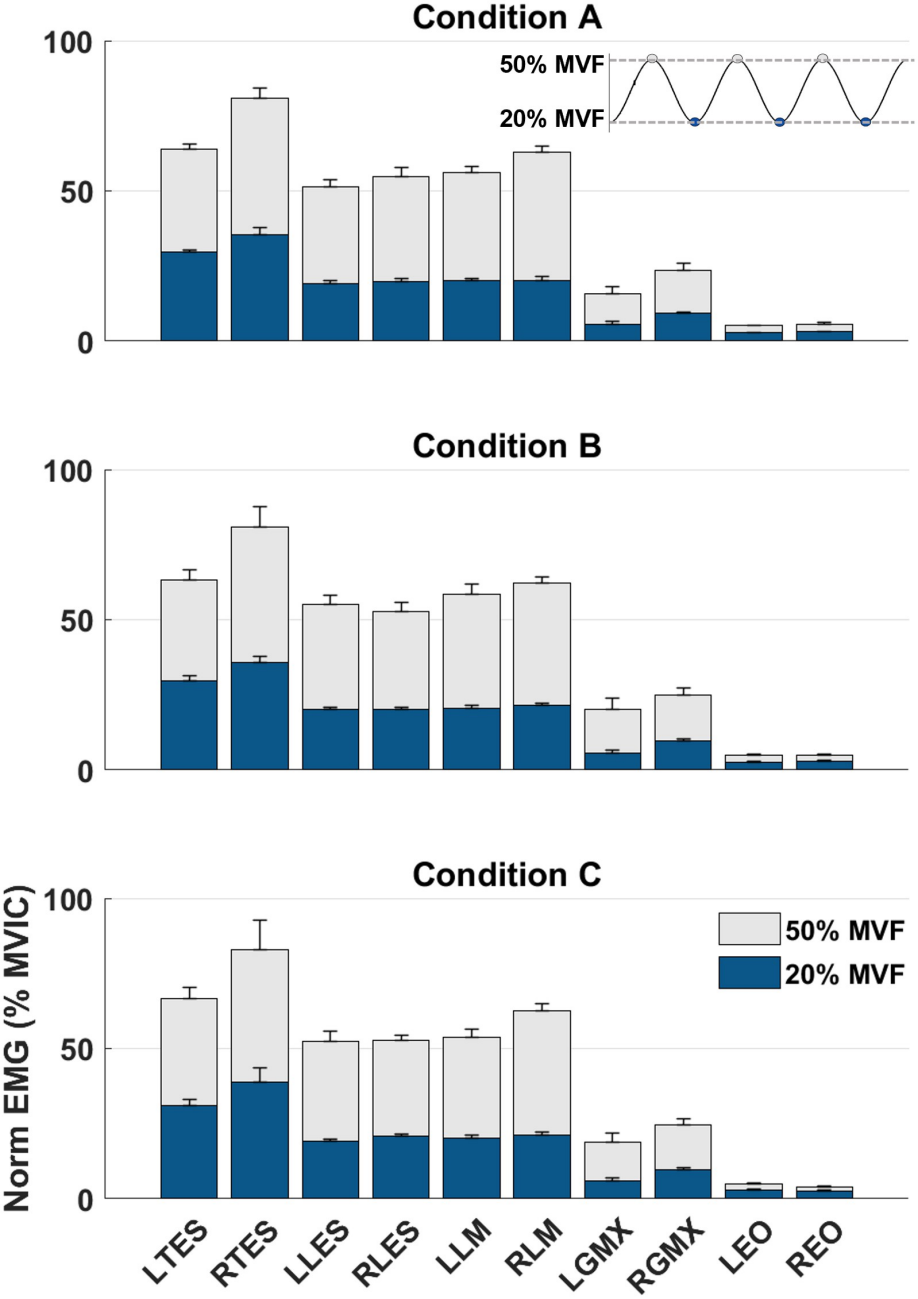
Mean amplitude normalized sEMG activation (+ SD) at minimum (20% of MVF, blue) and maximum target forces (50% of MVF, gray) during the force modulation task. EMG data were averaged across repetitions and participants. Right-sided muscles visually tended to demonstrate slightly more activation than left-sided muscles. MVF, maximal voluntary force; MVIC, maximal voluntary isometric contraction; LTES, left thoracic erector spinae; RTES, right thoracic erector spinae; LLES, left lumbar erector spinae; RLES, right lumbar erector spinae; LLM, left lumbar multifidus; RLM, right lumbar multifidus; LGMX, left gluteus maximus; RGMX, right gluteus maximus; LEO, left external oblique; REO, right external oblique.

**Fig 8:**
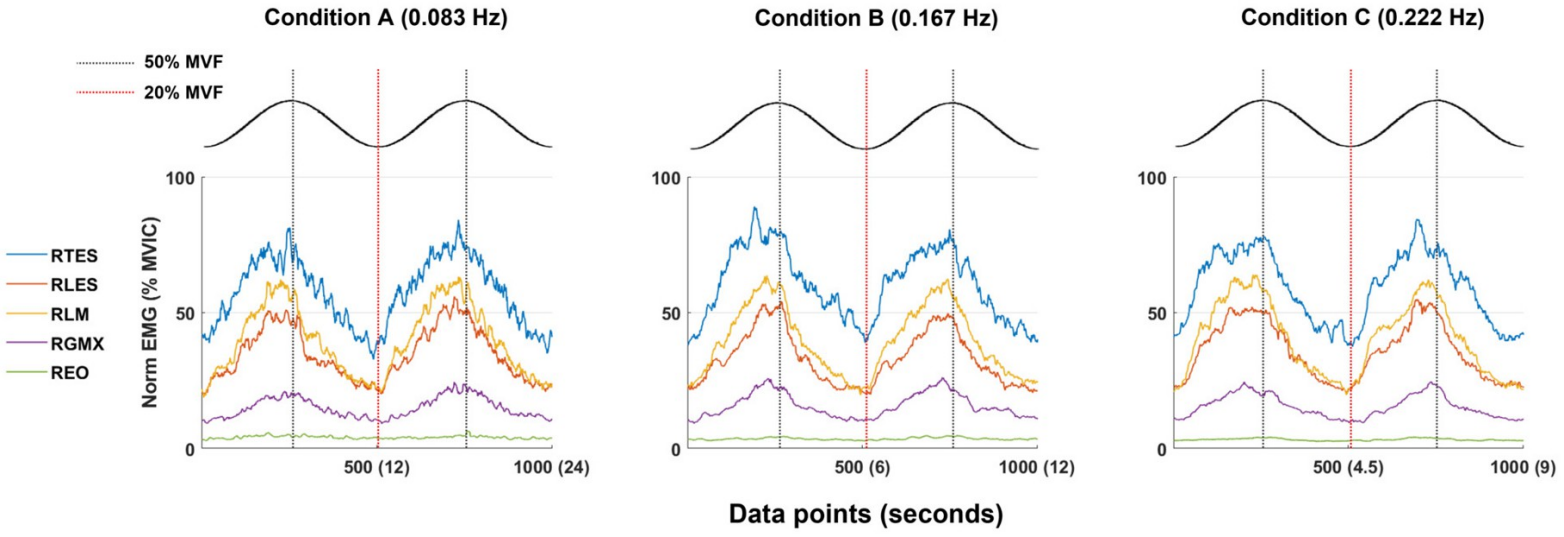
Mean time-series normalized muscle activation patterns during two modulation repetitions of each condition. Each repetition represents 500 data points. Only right-sided muscles are presented in this figure (left-side muscles show a similar pattern). The right external oblique muscle (REO) is minimally active across conditions. All other muscles appear to contribute to the force modulation. MVF, maximal voluntary force; MVIC, maximal voluntary isometric contraction; RTES, right thoracic erector spinae; RLES, right lumbar erector spinae; RLM, right lumbar multifidus; RGMX, right gluteus maximus.

The group averaged (n = 7) normalized sEMG amplitude at minimum (20% of MVF) and maximum target forces (50% of MVF) is presented in Fig 7. Regardless of condition, the left and right TES showed the highest activation at both 20% and 50% of MVF, followed by the activation of the left and right LM, left and rightLES, and left and right GMX. The left and right EO muscles showed the least activation in all three conditions and did not modulate or significantly contribute to the task. Fig 8 shows group-averaged amplitude normalized time series data of the muscle groups across two consecutive repetitions of each condition. Except for the EO, the muscles demonstrate an activation pattern mirroring the sine wave function used to modulate the force target with similar timing of peaks and troughs.

### Muscle synergy analysis

**Fig 9:**
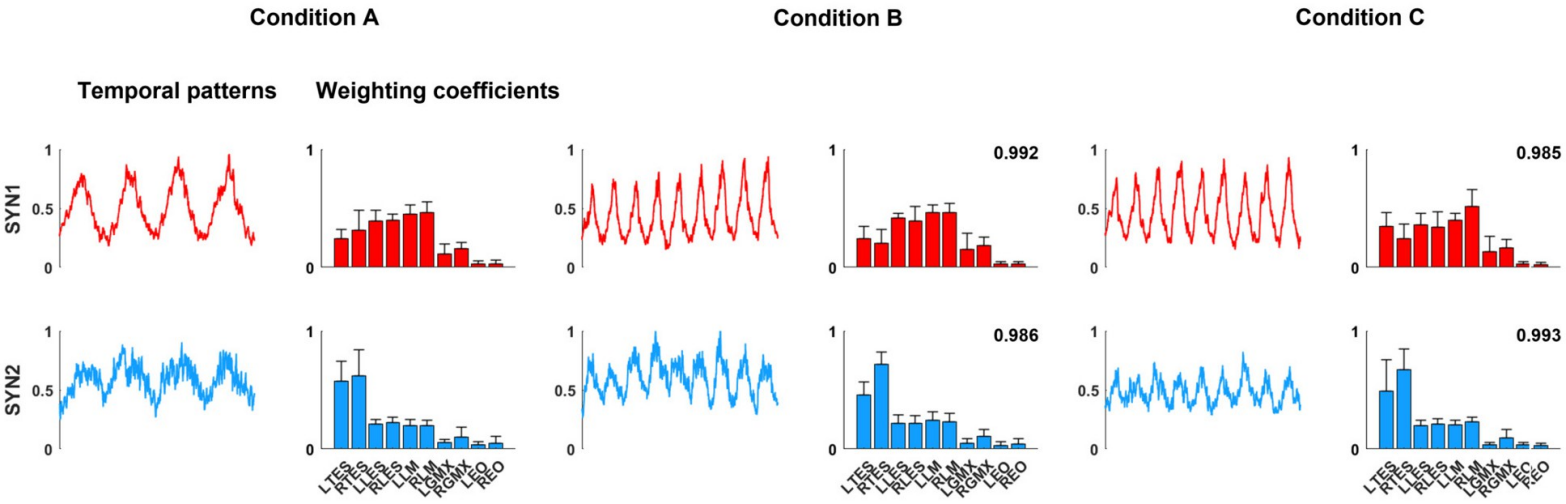
Group averaged muscle synergies extracted by non-negative matrix factorization. Temporal patterns and weighting coefficients (+SD) for three different conditions. Synergies presented in the same color between conditions indicate the same synergies with the mean scalar product greater than 0.85. The scalar products calculated compared to the synergies in condition A are presented on the graphs of conditions B and C, indicating the similarity between those synergies across conditions. SYN, synergy; LTES, left thoracic erector spinae; RTES, right thoracic erector spinae; LLES, left lumbar erector spinae; RLES, right lumbar erector spinae; LLM, left lumbar multifidus; RLM, right lumbar multifidus; LGMX, left gluteus maximus; RGMX, right gluteus maximus; LEO, left external oblique; REO, right external oblique.

Two to three synergies were extracted from individual participants for each condition. Weighted coefficients and temporal activations from the group-averaged extracted synergies are shown in Fig 9. Our clustering analysis identified two synergy patterns (SYN1-2) consistent across the three conditions that explained over 90% of the variance in the original EMG dataset. SYN1 was present in all seven participants and all conditions. SYN2 was present in 71% (5 out of 7) of participants in condition A and 86% (6 out of 7) in conditions B and C. The mean scalar product of the weighting coefficient vectors across participants ranged from 0.85 to 0.97, demonstrating a high similarity of synergies extracted for each participant and condition. Weighting coefficients and temporal activations of SYN1 and SYN2 were consistent across conditions. In general, SYN1 is responsible for the activation of the bilateral LM, LES, TES and, to less of an extent, activation of GMX. SYN1’s temporal activation pattern demonstrates a distinct sinusoidal pattern with large amplitude modulation and peak activation roughly coincides with peak target force across all three conditions. SYN2 was dominated by the activation of bilateral TES with more contribution from the right TES. The temporal activation pattern of SYN2 also shows sinusoidal modulation, but the amplitude of the modulation was less than SYN1. Activation peaks for SYN2 occurred near the peak target force, but slightly later than the peaks of SYN1. Overall, the EO contribution to both SYN1 and SYN2 was very small.

## Discussion

We sought to determine the test-retest reliability of trunk extensor FM accuracy and explore the construct validity of “trunk extensor FM” by using sEMG and NNMF. Results support our hypthesis that submaximal trunk extensor muscle FM accuracy can be reliably assessed. Our sEMG results reveal that known groups of trunk extensor muscles were indeed the muscles primarily used for FM during this task.

### Reliability

ICCs were uniformly excellent for both within-and between-day test-retest reliability, indicating that this protocol consistently quantifies trunk extensor FM accuracy and is a viable tool for future research. Not surprisingly, within-day test-retest values demonstrated higher ICC and lower SEM values compared with between-day The lowest ICC values were associated with the slowest rate of FM (condition A) and the highest ICCs were associated with the fastest rate (condition C). Condition C induced larger between-subject variability, which contributes to the higher ICC. Correlation coefficients benefit from moderate between-subject variability. The ICC and SEM carry complementary information, where ICC informs about the ability to rank subjects and SEM (as well as MDD) represents the precision of the measurement and points to the capacity to detect changes over (for example) a rehabilitation program. In addition to having the highest ICCs, faster modulation rates are attractive for future studies because they appear to be more challenging and produce a greater range of task performance (Fig 6).

Established MDD_95_ values allow future investigations to interpret FM accuracy change scores from this protocol. For example, if a researcher aimed to investigate the within-session effects of spinal manipulation, dry needling, or acute induced pain on FM accuracy using this protocol, they would need to observe a change in FM accuracy >2.4–3.2 RMSE to confidently attribute the change to the experimental stimulus. Similarly, if an investigator wanted to test a 1-week movement-based intervention aimed at ameliorating deficits in FM accuracy, improvements >3.4–4.9 N would be required to be confident these changes exceeded measurement error.

Pranata et al. [22] reported ICCs and SEMs from unpublished data for their trunk extensor FM protocol. Their parameters were identical to condition A in this study, except for the testing apparatus. For healthy participants (n = 16), they reported ICC = 0.88 and SEM = 0.5, and for chronic LBP participants, (n = 17) they reported ICC = 0.88 and SEM = 0.4. While not explicitly stated it is assumed, based on a single measurement session in their study design, that these values indicate within-day reliability. Reeves et al. [40] reported ICCs ranging from 0.99–1.00 and SEMs ranging from 0.2–0.44 for between-day trunk muscle FM accuracy reliability. Hadizadeh et al. [39] reported ICC ranging from 0.70–0.99 and SEMs ranging from 0.003–0.036 for within-day test-retest reliability for control error during their FM protocol. However, further comparison with the Hadizadeh et al. findings is not possible as the full-text is in Persian with only the abstract being published in English. The present study found within- and between-day ICCs that are higher than those reported by Pranata [22], and slightly lower than those reported by Reeves [40]. The SEMs from the present investigation were larger than previously reported values. Differences in ICC, and subsequently SEM, values could be attributed to differences in the ICC model selected. Pranata et al. [22] did not report the model used for their ICC calculations, while Reeves et al. [40] reported using model (3,k). Another factor influencing ICC differences may be more between-subject variability for our FM protocol. This increase in variation in our sample may be due to the increased number of participants demonstrating a wider array of FM abilities. Differences could also be attributed to differences in testing apparatus, intensity ranges, waveforms, and rates of FM.

There was no significant difference in RPE between testing blocks in either session, suggesting the potential confounding effect of fatigue was well controlled. For conditions A and B we observed differences in RPE over time, where participants rated the first block on the second day as less exertion than one or both of the testing blocks on the first day. This decrease in exertion could be the result of familiarization with the task. Effect sizes (Cohen’s d) for differences in RPE between the second block of the first day and the first block of the second day were moderate (d = 0.60) and small (d = .47) for conditions A and B, respectively [55]. Additionally, we calculated between-session MDD_95_ for the Borg RPE scale from reliability data from Lamb et al. [46] and found that a change of around 3 would be needed to detect a true difference using this scale. This may be an example of statistically significant findings that are not meaningful.

### Validity

Our data indicate that the bilateral TES has the highest mean activation amplitude for all conditions. This may be influenced by the positioning of the pulley at the height of the xiphoid process and the location of electrode placement for TES (at the level of the T9 spinous process). While the largest amplitudes were observed in the trunk extensor muscles (TES, LES, LM), GMX, a hip extensor and hip external rotator, also demonstrated increases in amplitude up to 25% MVIC. While our goal was to minimize hip extension movement and the contribution of GMX to force modulation, we expected increased activation of this muscle group to some extent. With the femur immobile on the chair, GMX would act on the pelvis with an internal extensor moment and contribute to the trunk (lumbopelvic) stiffness needed for task performance.

Where the mean amplitude tells us about the activation increase of individual muscles, NNMF delineates the neuromuscular control strategy implemented and how muscles interact within this task. SYN1 demonstrates the greatest modulation amplitude and is dominated by the weightings of LM and LES. TES and GMX also contribute to SYN1, but less. This evidence indicates GMX is responsible for some FM. SYN2 is dominated by the TES, suggesting that the TES is primarily driven by a separate synergy with slightly different timing in this task. The dominance of right versus left TES in SYN2 is difficult to interpret. This could be related to handedness, as previous work found differences in trunk muscle recruitment strategies based on handedness during an arm lifting task [56]. Additionally, the experimental setup did not constrain trunk rotation. SYN2 demonstrates a temporal activation pattern similar to SYN1 but with less amplitude of modulation and a slightly delayed peak amplitude. Across FM conditions SYN1 and SYN 2 were not influenced by the modulation rate. The similarity in synergy patterns is not surprising given the task is the same [57]. Our synergy analysis supports the construct validity of “trunk extensor” FM, with the acknowledgment that the GMX contributed some to SYN1 and the modulation of the force during the task.

We found a negative correlation between physical activity (MLTPAQ) and FM error, meaning that individuals with higher physical activity levels performed better on these tasks. We believe this finding additionally supports the construct validity of these FM tasks. It is well documented that acute and chronic physical activity positively impact cognitive performance [58], which is a component of our FM task. A systematic review of 19 studies comprised of 15,984 participants supported a moderate to large positive relationship between physical fitness and motor control in children and young adults, and this relationship strengthened with age [59]. Most relevant to this research, a study by Stodden et al. [60] on a sample of 188 young adults ages 19–25 found that greater motor skill competence predicted better physical fitness.

### Limitations

A limitation of this work is that these findings are not generalizable to individuals with LBP [61]. Individuals with LBP have more variable muscle performance and motor control [62, 63] and pain resulting from musculoskeletal disorders that can influence muscle synergies [64]. On the other hand, the unpublished data by Pranata et al. [22] indicates similar ICCs and SEMs for FM accuracy in both a healthy cohort and individuals with LBP. Future studies should confirm reliability in this population. Our inclusion criteria restricted the age of participants to 18–35 years, rendering these findings not generalizable to older individuals as age has been shown to affect trunk muscle synergies [65]. In the present investigation, we experimentally manipulated only the rate of FM and found slight differences in conditions based on this parameter. It would be worthwhile to determine how other parameters such as intensity range for FM (%MVF), modulation waveform, and the presence or absence of feedback on participant-generated force influence FM accuracy and the utility of these protocols. Additionally, we did not randomize the order of the conditions or control for the time of day of testing, both of which could have influenced our FM accuracy values. However, we did not find differences in fatigue (RPE) across conditions. The addition of a more complete set of trunk and hip muscles, especially muscles like the hamstrings and other abdominals, and additional subjects may provide a more comprehensive picture of the synergies employed during this task.

## Conclusions

Within- and between-day test-retest reliability for FM accuracy was uniformly excellent across the three conditions tested. Conditions with faster rates of FM demonstrated higher ICCs while inducing more error and larger between-subject variability. sEMG demonstrates that this protocol elicits the greatest muscle activity from the TES, LES, and LM. NNMF reveals a consistent control strategy across participants and conditions where the modulation of force was dominated by the trunk extensors.

## Supporting information

S1 Appendix sEMG collection details. (DOCX)

S1 Checklist (PDF)

## Abbreviations

EO: external oblique
FM: Force modulation
GMX: gluteus maximus
ICC: Intraclass correlation coefficient
LBP: Low back pain
LES: lumbar erector spinae
LM: lumbar multifidus
MDD: Minimal detectable difference
MVF: maximal voluntary force
MVIC: Maximal Voluntary Isometric Contraction
NNMF: Non-negative matrix factorization
RMSE: Root mean square error
RPE: Rate of perceived exertion
SEM: Standard error of measurement
sEMG: surface EMG
TES: thoracic erector spinae
VAF: variance accounted for
