# Supplementary material for: Test-retest reliability and construct validity of trunk extensor muscle force modulation accuracy
Source: PLoS One. 2023 Aug 17;18(8):e0289531. doi: 10.1371/journal.pone.0289531 (PMC10434934; doi:10.1371/journal.pone.0289531)
Supplement: S1 Appendix — (DOCX) [file pone.0289531.s001.docx]

**S1 Appendix**. **sEMG collection details**

**Delsys Trigno sEMG sensors (Delsys, Natick, MA)**

- Active, bipolar, differential electrodes with parallel bar sensors
- 5x1 mm contact dimension
- 10-mm interelectrode distance
- CMRR >80 dB
- bandwidth 20–450 Hz

**sEMG sensor placement**

Figure. **sEMG sensor placement.** Placement of surface electromyography sensors. Electrode locations were standardized and consistent with prior literature [38-40]. External oblique sensors applied anteriorly on the lateral abdominal wall.


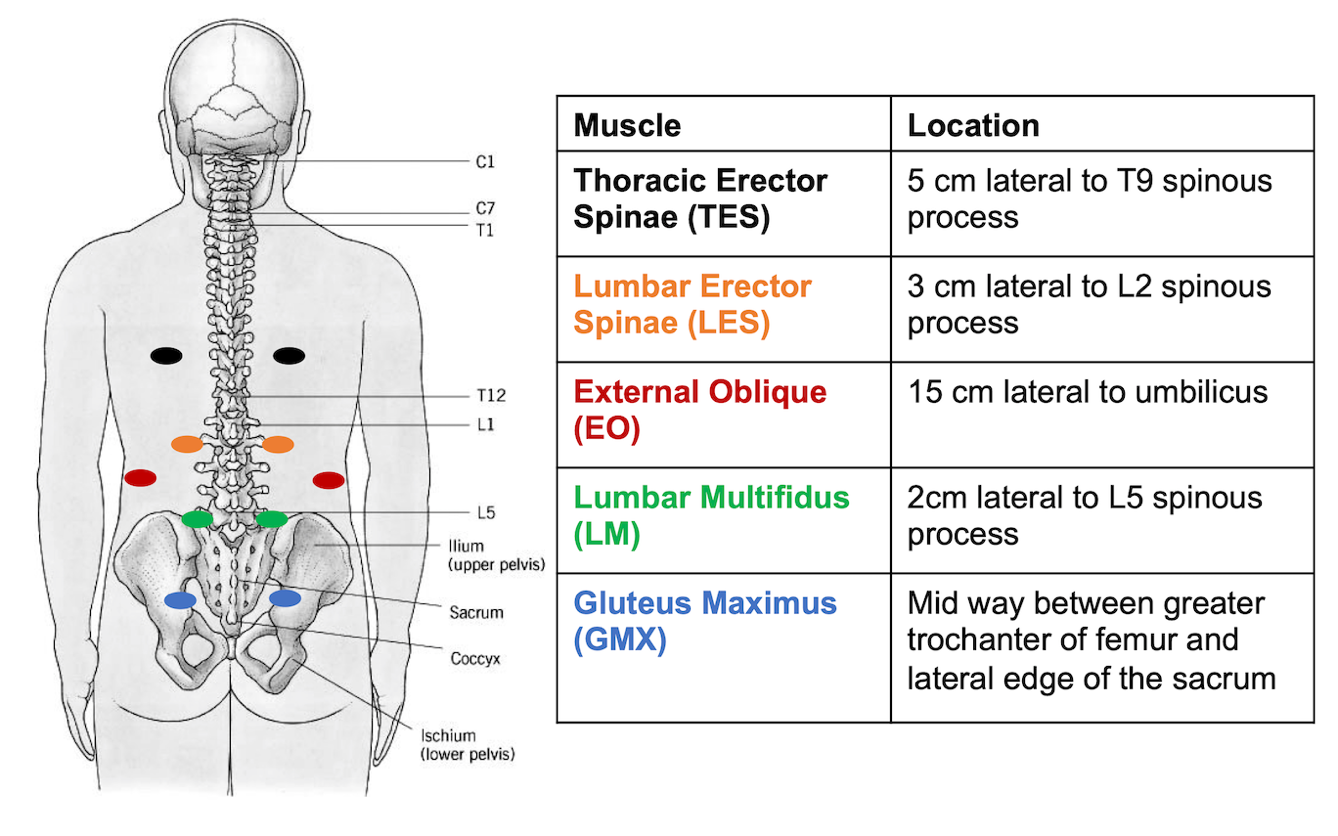


**Individual muscle MVIC**

Our testing apparatus is designed to bias muscle activation and force production to the trunk extensor muscles. To normalize sEMG values we collected individual muscle maximal voluntary isometric contractions (MVICs) for the gluteus maximus (GMX) and external oblique (EO). Gluteus maximus was tested by manually resisting hip extension in standing with the trunk supported on a table at the height of the pubic symphysis. Manual resistance was applied to the plantar surface of the foot with the hip flexed, knee flexed to 90°, and ankle in a neutral position. The external oblique muscles were tested by performing an abdominal crunch in hook lying and resistance was provided by the examiner into contralateral rotation and extension. Each test was performed bilaterally. Following these MVIC trials, participants were then positioned in the testing apparatus and MVICs for the trunk extensor muscles were collected during the maximum voluntary extensor force (MVF) protocol.
